# Shaping the future of psychotherapy: Leveraging technology-supported online clinics

**DOI:** 10.1192/j.eurpsy.2025.10118

**Published:** 2025-10-16

**Authors:** Franziska Miegel, Anna Brähler, Lara Wille, Amir H. Yassari, Lara Rolvien

**Affiliations:** Department of Psychiatry and Psychotherapy, Universitatsklinikum Hamburg-Eppendorf, Hamburg, Germany

**Keywords:** Digital mental health innovation, Virtual psychotherapy, Extended reality (XR) interventions, Scalable care models, Access to intensive treatment

## Abstract

Melanie, a mother of two, has struggled with severe contamination-related obsessive-compulsive disorder (OCD) for years, spending hours each day cleaning and avoiding activities that feel “unsafe,” which causes constant anxiety. Her symptoms make daily tasks difficult, as she cannot play with her children outside, do groceries without taking drastic measures, or visit friends and family without intense fear of contamination. Her rigid routines and compulsions take a toll on her mental and physical health and her children’s, leaving her feeling isolated and overwhelmed. Melanie knows she needs specialized treatment. However, living in a rural area, the nearest specialized clinic is too far away for a day treatment program, inpatient care is not an option due to childcare responsibilities, and outpatient treatment is inaccessible due to long waiting lists and insufficient intensity for her needs. As a result, she remains stuck, unable to access the intensive therapy she requires.

Melanie, a mother of two, has struggled with severe contamination-related obsessive-compulsive disorder (OCD) for years, spending hours each day cleaning and avoiding activities that feel “unsafe,” which causes constant anxiety. Her symptoms make daily tasks difficult, as she cannot play with her children outside, do groceries without taking drastic measures, or visit friends and family without intense fear of contamination. Her rigid routines and compulsions take a toll on her mental and physical health and her children’s, leaving her feeling isolated and overwhelmed. Melanie knows she needs specialized treatment. However, living in a rural area, the nearest specialized clinic is too far away for a day treatment program, inpatient care is not an option due to childcare responsibilities, and outpatient treatment is inaccessible due to long waiting lists and insufficient intensity for her needs. As a result, she remains stuck, unable to access the intensive therapy she requires.

Melanie’s story is not unique; it reflects the reality of many patients who fall through the cracks of our healthcare systems. Neither in Germany, where Melanie lives, nor in many other countries do existing structures adequately reach those who require specialized, intensive treatment but face geographical, logistical, or systemic barriers to access. Around the world, patients face long waiting times – those in Germany, for instance, wait nearly 5 months on average for outpatient treatment [1]. In many cases, inpatient therapies are simply out of reach due to a combination of structural barriers, such as limited funding, insufficient availability, long travel distances, and personal circumstances, including caregiving responsibilities for children or elderly parents [2]. Additionally, even when patients are hospitalized, they often receive inadequate psychotherapy. A meta-analysis revealed that, on average, inpatients only receive about 60 min of psychotherapy per week [3], despite evidence showing that more intensive therapy, such as doubling the frequency of sessions, is linked to significant improvements in symptoms [4].

The lack of a comprehensive treatment model that integrates both flexibility and intensity is a critical gap in mental health systems worldwide. Mental disorders remain frequently undertreated, particularly among patients who are bound to their homes due to caregiving responsibilities or other obligations and, therefore, do not receive adequate support, leading to far-reaching consequences, including social isolation, suicidal ideation, and severe financial and personal difficulties for those affected [5]. While exact numbers are difficult to obtain due to underreporting and lack of systematic outreach, estimates based on prevalence data and current treatment gaps suggest that several hundred thousand individuals in Germany alone, many of whom are currently underserved, could benefit from such a model each year. Capturing this “invisible population” remains a key challenge, underscoring the urgent need for structured research and digital health innovation.

What’s needed is a fundamentally new approach to psychiatric care, one that bridges these gaps and reaches the patients who have long been left behind. A technology-supported online clinic could be the answer, merging the frequency and intensity of inpatient care with the flexibility and accessibility of remote treatment. For patients like Melanie, who face the compounded challenges of distance, childcare responsibilities, and long waiting times, this model offers the possibility of receiving evidence-based therapy tailored to their unique circumstances without the need to sacrifice family life or travel long distances. By bringing therapy directly into patients’ homes, it addresses the logistical barriers and the need for high-intensity care, offering a lifeline to those in rural or underserved areas who otherwise may never receive the care they need.

Beyond traditional teletherapy, often limited to verbal sessions, a technology-supported online clinic offers a comprehensive, immersive therapeutic experience that replicates, and in many ways improves upon, almost the full spectrum of inpatient care. The approach we envision goes beyond simple video calls by incorporating controlled, virtual environments that support evidence-based therapies. Patients can engage in real-time therapeutic exercises within these immersive settings, addressing their specific challenges more dynamically and personally. What sets this model apart is its ability to integrate almost all inpatient program components into daily virtual and non-virtual sessions, including individual therapy, psychoeducation, mindfulness training, group therapy, music therapy, and physical exercises. See Figure 1 for a comprehensive overview of all treatment elements demonstrated in a potential treatment schedule.

**Figure 1.**
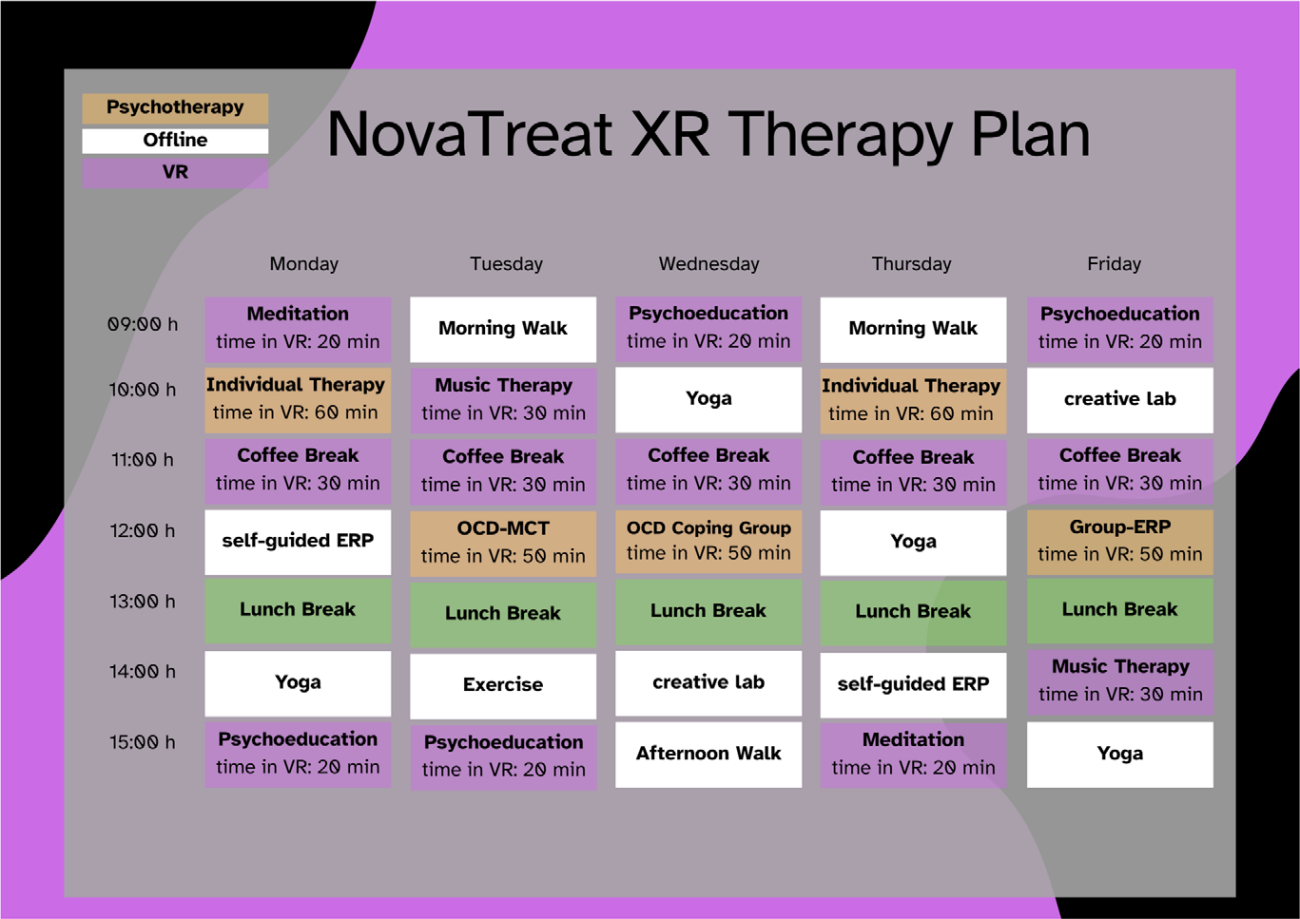
Potential treatment plan for OCD, including all therapy elements. *Note.* Psychotherapy modules (orange) and other extended-reality (XR) modules (purple) can encompass both virtual- and mixed-reality formats. The modules include an estimated time spent in XR to ensure that patients do not wear the XR headset too long, thereby preventing cybersickness or pressure marks from the glasses. Psychotherapy modules include individual therapy, metacognitive training (MCT-OCD) group sessions, OCD coping group sessions, and exposure and response prevention groups (group-ERP). Modules held in XR include meditation, a time in which patients can interact with other patients in a virtual break room (coffee break), psychoeducation, and music therapy. Offline modules (white) include self-guided exposure and response prevention exercises (ERP), yoga, walks, physical exercise, and artistic and expressive tasks (creative lab).

A comprehensive library of Artificial Intelligence (AI)-based video tutorials created by therapists will be developed to provide psychoeducation on key elements, such as planning positive activities, establishing daily routines, preparing for exposures, and much more. This approach ensures that patients receive high-quality, standardized psychoeducation and helps conserve therapeutic resources by shifting these foundational components out of individual therapy sessions. As regulatory frameworks for therapeutic use of AI continue to evolve, the integration of virtual assistants is also planned to further optimize resource allocation, support patients in self-guided therapeutic activities, and help therapists adjust the treatment plan to be most effective (clinical decision support systems augmented with AI). All these interventions, which are typically spread out over time in traditional settings, will be delivered daily, optimizing therapeutic outcomes. AI enables resource efficiency, allowing therapists more time to focus on individualized, intensive psychotherapy. Consequently, the overall duration of therapy may be significantly reduced, for example, from 10 to 5 weeks. The approach will lead to substantial savings in room and meal costs, therapist availability, and group session capacity, all while preserving therapeutic depth.

Furthermore, many elements traditionally confined to inpatient programs, such as physical activity, meditation, and yoga, can be instructed daily via virtual avatars or videos and performed in reality (without glasses). Research has already shown the positive impact of regular movement and mindfulness on mental health, and this daily integration is crucial for sustaining long-term recovery [6]. In addition, virtual group therapy can be a cornerstone of the treatment model, replicating the positive social aspects of inpatient care. Online coffee breaks and informal virtual meetups facilitate peer interaction, ensuring that the social benefits of group therapy and the social aspects of inpatient treatment remain intact, even in a remote setting. Since patients cannot wear the XR headsets for 8 h a day and the transferability and practice of skills in the here and now is one of the most crucial aspects of therapy, the focus will still be on doing as much as possible in real life, such as self-guided exposures, implementing positive activities, or engaging in physical exercise that is planned in XR and conducted in real life. This combination of intensive psychotherapy, daily therapeutic activities, and regular group support makes the technology-supported online clinic a unique and holistic solution for mental health care, offering a level of care that is more accessible, intensive, and flexible than what is currently available in traditional psychiatric systems.

In recent years, research has shown that virtual- and mixed-reality interventions can not only improve treatment outcomes for various disorders, including depression, anxiety, and OCD, but also enhance other non-clinical domains like mindfulness [7, 8]. Until now, there has been no collective platform for accessing and utilizing various developed virtual psychological interventions. The XR-online clinic seeks to bridge this gap by integrating scientifically proven therapies into a novel digital infrastructure for psychological care, complementing traditional inpatient and outpatient treatments. A current project funded by the German Federal Ministry of Research aims to develop a software to create an XR environment for this purpose. More information can be found on the funder’s website (https://www.interaktive-technologien.de/projekte/stivok and this website: https://novatreat.de/). In the long term, this initiative seeks to expand healthcare structures worldwide, making innovative psychological care more accessible and practical.

However, there are certain limitations to consider. Patients will still need to visit a specialized center before and after the treatment period for physical examinations and technical setup. Additionally, not all psychiatric conditions are suitable for technology-supported interventions. Patients experiencing severe suicidality, acute psychosis, or cognitive impairments may still require traditional inpatient care or highly supervised treatment, as technology-supported online clinics may not provide the necessary level of support for these cases. Of course, also for all other types of disorders and patients, a sophisticated emergency plan must be integrated for times of acute crises or sudden suicidality. In the future, online clinics could potentially be connected to the general emergency care infrastructure (e.g., fire department, emergency medical services, and sector hospitals) to ensure the fastest possible assistance. While data security and patient privacy questions are critical, these challenges can largely be mitigated by hosting sensitive health data on secure, internal clinic servers and ensuring that patient participation occurs via encrypted connections, even though patients access the platform through their home networks. Clear consent procedures, compliance with medical data regulations, and transparent handling of digital interactions are essential.

Despite the potential of technology-supported online clinics, a “know-do gap” exists, preventing these innovations from reaching widespread clinical practice. While research demonstrates the effectiveness of technology-supported interventions in treating conditions like anxiety, post-traumatic stress disorder, and OCD, these therapies have not yet been integrated into everyday care due to systemic barriers such as inadequate funding, infrastructure, and policy support. Overcoming these obstacles is crucial to ensure that these therapies reach those who need them most [9, 10]. Another key aspect is developing and integrating the necessary infrastructure into existing hospital systems. This includes incorporating services into current outpatient and inpatient settings and creating specialized departments for technology-supported online therapy. While this requires upfront investment, leveraging existing organizational structures can make the implementation more efficient. This not only enhances flexibility for patients but also benefits healthcare providers by enabling clinical care from home. Such a model could improve work-life balance, particularly for women, who still bear the majority of family responsibilities, thereby contributing to greater gender equality.

Ultimately, the vision outlined here is not just about technological innovation but about rethinking the very structure of psychiatric care. It is about creating a system that is more accessible, more personalized, and more sustainable, one that is better equipped to address the complex needs of patients across the globe. This shift is not a distant possibility, but an urgent necessity that can transform lives, reduce disparities, and contribute to a more equitable and effective mental healthcare system worldwide.
